# Development and interpretability of a machine learning-derived model for the identification of latent prostate cancer in patients initially diagnosed with benign prostatic hyperplasia: a retrospective cohort study

**DOI:** 10.1186/s12911-026-03623-w

**Published:** 2026-06-08

**Authors:** Sheng Liang, Yuansi Lao, Peiling Ma, Changhuan Shang, Rukun Li, Zengfu Li, Yongtao Le, Wenhui Cao, Chunling Chen, Ke Liu, Liqing Li

**Affiliations:** 1 Department of Clinical Laboratory, Hengzhou City People’s Hospital, Hengzhou, Guangxi China; 2 Department of Radiation Oncology, Guangxi Medical University Cancer Hospital, No.71, Hedi Road, Nanning, China

**Keywords:** Prostate cancer, Benign prostatic hyperplasia, Machine learning, Model interpretability, Biomarkers, Prostate-specific antigen, SHAP, Risk stratification

## Abstract

**Background:**

Prostate cancer (PCa) is characterized by pronounced intratumoral heterogeneity and multifocality, presenting ongoing challenges for early and precise diagnosis. In clinical practice, a substantial proportion of patients initially diagnosed with benign prostatic hyperplasia (BPH) are subsequently found to harbor latent PCa during follow-up. However, current prostate-specific antigen (PSA)-based screening lacks the specificity required to resolve this diagnostic overlap. This study aimed to develop and internally validate an interpretable machine learning (ML) model as an adjunct to PSA testing for identifying latent PCa in patients initially diagnosed with BPH.

**Methods:**

This retrospective cohort study utilized data from 1,071 patients to develop a machine learning framework aimed at identifying pre-existing occult PCa diagnosed at least 6 months after the initial BPH assessment. The cohort was partitioned into training (70%) and test (30%) subsets. Within the training set, Recursive Feature Elimination (RFE) was utilized to identify a concise biomarker signature. Six machine learning algorithms were evaluated: logistic regression (LR), random forest (RF), extreme gradient boosting (XGB), support vector machine (SVM), k-nearest neighbors (KNN), and multilayer perceptron (MLP). Model performance was assessed using the area under the receiver operating characteristic curve (AUROC), Brier score, and decision curve analysis (DCA). Model interpretability was facilitated through SHapley Additive exPlanations (SHAP) and Locally Weighted Scatterplot Smoothing (LOWESS) analysis.

**Results:**

A concise four-biomarker signature comprising total prostate-specific antigen (tPSA), lactate dehydrogenase (LDH), red blood cell count (RBC), and creatine kinase-MB (CK-MB) was identified. The MLP and LR models exhibited high diagnostic performance, with test AUROC values of 0.949 and 0.946, respectively.

**Conclusion:**

This research developed and internally validated an interpretable machine learning framework designed to complement traditional PSA screening methodologies. The tool is intended exclusively for research purposes.

## Introduction

Prostate cancer (PCa) remains a leading cause of oncological morbidity and mortality in men globally [1]. Diagnostic evaluation is complicated by the inherently multifocal nature and genomic diversity of the disease, which frequently involves androgen receptor (AR) signaling alterations, PTEN loss, and TP53 mutations [2–4]. In clinical settings, a substantial number of patients initially diagnosed with benign prostatic hyperplasia (BPH) are found to harbor latent PCa during subsequent follow-up. This diagnostic lag, in which pre-existing occult cancer is overlooked at the initial BPH assessment, underscores the limitations of prostate-specific antigen (PSA) as a standalone biomarker, particularly its low specificity in distinguishing concurrent benign and malignant prostatic conditions [5, 6].

The transition from conventional linear statistical modeling to advanced machine learning (ML) architectures offers an opportunity to capture complex, non-linear biological interactions often missed by traditional analyses [7, 8]. Although preclinical models, including cell lines, patient-derived xenografts, and organoids, have significantly advanced the understanding of PCa genomics [9, 10], clinical diagnosis remains reliant on peripheral blood biomarkers. However, the integration of routine hematological and biochemical markers into a comprehensive diagnostic framework remains insufficiently explored [8]. Recent developments in knowledge-assisted hybrid modeling demonstrate that incorporating structural clinical insights can mitigate the “curse of dimensionality” inherent in clinical datasets, thereby improving model generalizability [11]. Furthermore, such knowledge-augmented frameworks are essential for ensuring that predictive tools maintain robustness and generalizability across diverse clinical settings [12].

Despite these analytical advantages, the opaque nature of artificial intelligence has historically limited its clinical adoption. An effective diagnostic tool must be both accurate and interpretable, providing clinicians with biologically plausible explanations [13, 14]. In this study, we addressed these challenges by developing an interpretable machine learning framework. Utilizing a longitudinal cohort of 1,071 patients, we applied Recursive Feature Elimination to determine an optimal set of routine markers and subsequently validated six machine learning models. This framework is conceptually designed to capture the systemic and microenvironmental signatures of latent tumors rather than to predict de novo carcinogenesis. We integrated SHAP (SHapley Additive exPlanations) to resolve model decisions, thereby aligning computational outputs with established pathological mechanisms to ensure clinical transparency [14, 15].

## Materials and methods

### Study population

In this retrospective cohort study, we enrolled 1,071 male patients who presented with lower urinary tract symptoms (LUTS) and received an initial clinical diagnosis of BPH at our institution. The diagnosis was established using a standardized diagnostic protocol that included digital rectal examination (DRE), transrectal ultrasound (TRUS), and assessment with the International Prostate Symptom Score (IPSS). Eligible participants had available baseline records for 22 clinicopathological variables, including total prostate-specific antigen (tPSA), lactate dehydrogenase (LDH), creatine kinase-MB (CK-MB), and red blood cell count (RBC). To ensure data integrity, patients with a history of malignancy, active prostatitis within the preceding 3 months, or active treatment with 5-alpha reductase inhibitors (5-ARIs) were excluded. To identify latent malignancies, all patients underwent a standardized longitudinal follow-up protocol, including semi-annual monitoring of PSA levels and clinical reassessment. The primary outcome was histologically confirmed PCa diagnosed at least 6 months after the initial BPH assessment. The diagnosis of PCa was confirmed by histological analysis of specimens obtained from transrectal ultrasound (TRUS)-guided systematic 12-core biopsy or radical prostatectomy. During the study period, 111 incident cases of PCa (10.4%) and 960 cases of stable benign prostatic hyperplasia (BPH) (89.6%) were identified. The final analytical cohort comprised all 1,071 initially eligible patients, as no patients were excluded because of missingness after application of the prespecified variable-level missingness threshold and imputation strategy.

### Feature engineering and selection

Candidate variables with a missing data rate exceeding 30% were excluded prior to imputation. The rationale for this threshold is that while most variables in our dataset exhibited minimal missingness, a distinct subset of clinical parameters had disproportionately high missing rates. Excluding predictors with substantial missingness avoids the introduction of imputation-induced noise and bias, a practice recommended in recent methodological guidelines for developing clinical prediction models [16]. To empirically validate this threshold, a sensitivity analysis across varying missingness exclusion thresholds was subsequently performed. To rigorously prevent data leakage, the dataset was randomly divided into a training set (70%) and an independent test set (30%). After this partitioning, missing values were addressed separately within each set. For continuous variables, imputation was performed using a multivariate Iterative Imputer, configured with a maximum of 10 imputation rounds and a convergence tolerance threshold of 1e-3. Categorical variables were imputed using a Simple Imputer with a mode strategy.

To improve model generalization and mitigate the “curse of dimensionality,” we implemented Recursive Feature Elimination (RFE) exclusively within the training cohort. RFE is a robust technique for identifying the most stable predictors in multidimensional healthcare datasets [7, 17]. We employed a random forest classifier as the base estimator within a 5-fold cross-validation framework. To ensure reproducibility, hyperparameters for this estimator were defined with the number of trees (n_estimators) set to 100, and the maximum depth (max_depth) left unconstrained to allow nodes to expand until all leaves were pure. The optimal number of features was determined by observing the performance plateau of the mean Receiver Operating Characteristic (ROC) score across folds. Features with a normalized importance score greater than 0.1 that significantly contributed to the cross-validated Area Under the Curve (AUC) were retained, ensuring a parsimonious and highly stable predictive signature.

### Model training and cross validation

Six classifiers, including LR, RF, XGB, SVM, KNN, and MLP, were trained and evaluated. Hyperparameter optimization was conducted using GridSearchCV on the training set to maximize the Area Under the Receiver Operating Characteristic Curve (AUROC). To rigorously address overfitting and ensure unbiased performance estimates, three advanced internal validation strategies were employed: [1] nested cross-validation to distinctly separate the tuning and evaluation processes; [2] bootstrap optimism correction with 100 resamples to quantify and adjust for model optimism, defined as the discrepancy between apparent and test performance; and [3] repeated cross-validation, specifically 10 × 5-fold, to assess algorithmic stability across various data permutations. Additionally, model robustness in ambiguous clinical scenarios was assessed through subgroup analysis within the PSA gray zone (4.0 to 10.0 ng/mL). To address the class imbalance within this sub-cohort, the Synthetic Minority Over-sampling Technique (SMOTE) was incorporated into the cross-validation pipeline. This resampling strategy was used to reduce majority-class dominance during model training and to provide exploratory estimates of algorithmic performance under severe class imbalance.

### Model interpretability and clinical utility

The SHAP framework was employed to attain both global and local interpretability. The clinical net benefit was quantified via Decision Curve Analysis (DCA), facilitating a comparison between our machine learning framework and conventional screening strategies, namely “treat all” or “treat none”.

## Results

### Baseline characteristics of the study cohort

**Fig. 1:**
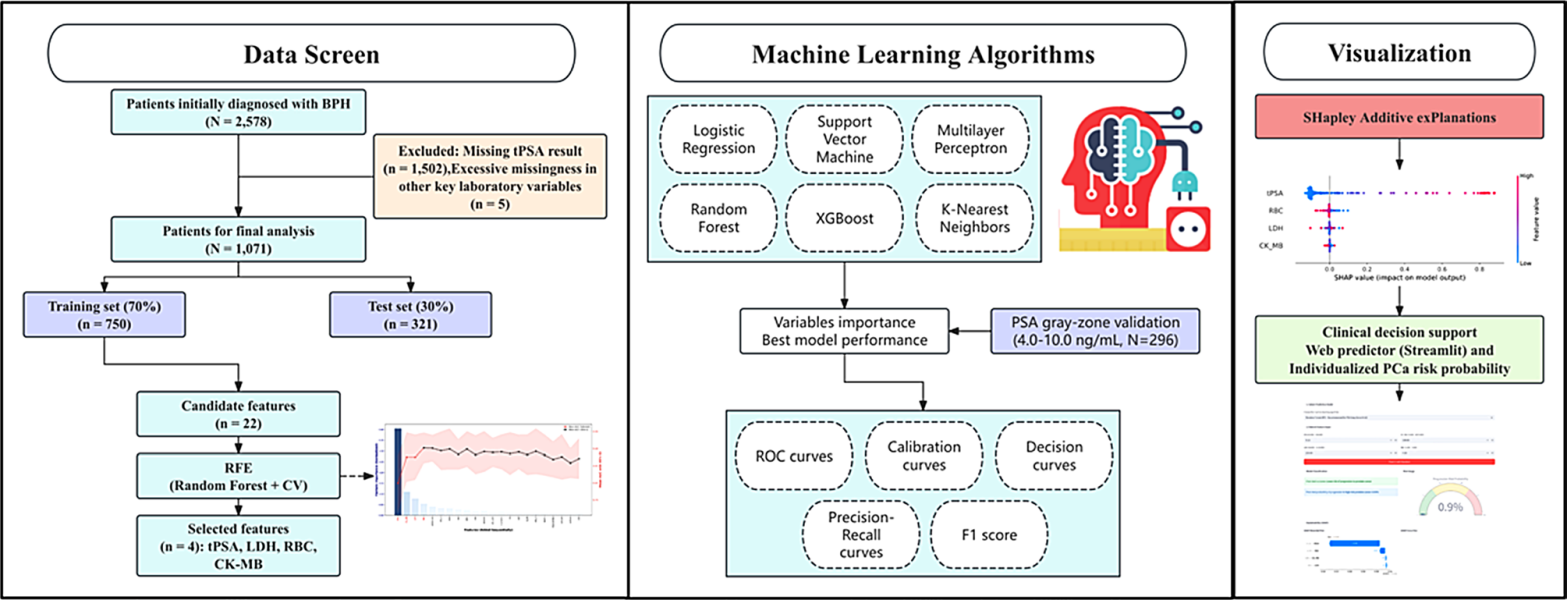
Flowchart of the overall study design

The comprehensive study design and patient flow are depicted in Fig. 1. This study encompassed a cohort of 1,071 patients initially diagnosed with benign prostatic hyperplasia (BPH). During a follow-up period of at least six months, 111 patients (10.4%) were found to harbor latent prostate cancer (PCa), while 960 patients (89.6%) remained stable. The baseline demographic and clinical characteristics of the cohort are detailed in Table 1. Patients diagnosed with PCa were significantly older, with a median age of 76.00 years (interquartile range [IQR]: 70.00 to 80.00), compared to 71.00 years (IQR: 65.00 to 77.00) in the stable group (*P* < 0.001). Additionally, these patients demonstrated significantly higher baseline tPSA levels (median: 80.96 ng/mL, IQR: 20.35 to 100.00) compared to the stable group (median: 4.49 ng/mL, IQR: 1.88 to 9.70; *P* < 0.001). Other biomarkers, including lactate dehydrogenase (LDH) and red blood cell (RBC) counts, also exhibited distinct trends between the two groups.

**Table 1:** Baseline demographic and clinical characteristics of the study cohort

Feature	Overall	Stable BPH	Incident PCa	P_Value
age	72.00 [66.00–77.00]	71.00 [65.00–77.00]	76.00 [70.00–80.00]	< 0.001
BMI	22.94 [21.01-25.00]	22.99 [21.05–25.08]	22.04 [20.71–24.19]	0.501
globulin	27.60 [24.80–30.60]	27.40 [24.70–30.50]	29.00 [26.10-31.45]	0.016
ALP	2.22 [2.11–2.31]	2.22 [2.12–2.31]	2.22 [2.09–2.32]	0.956
CK-MB	108.00 [73.10–160.00]	106.00 [72.75–157.00]	131.00 [89.50-175.80]	0.601
sodium	141.00 [138.80–144.00]	141.00 [138.80–144.00]	141.50 [138.65–144.00]	0.492
calcium	2.22 [2.11–2.31]	2.22 [2.12–2.31]	2.22 [2.09–2.32]	0.956
LDH	203.00 [175.00-241.00]	201.00 [173.00-239.00]	222.40 [193.00-274.50]	0.008
CK	141.00 [138.80–143.00]	141.00 [138.80–143.00]	140.80 [138.65-142.36]	0.712
creatinine	95.00 [78.00-129.00]	95.00 [78.00-128.25]	95.00 [78.00-129.00]	0.65
uric_acid	354.00 [283.00-432.00]	353.00 [282.00-434.77]	359.00 [294.50-420.95]	0.6
triglycerides	1.14 [0.82–1.67]	1.15 [0.82–1.70]	1.06 [0.80–1.43]	0.04
HDL_C	1.18 [0.98–1.42]	1.19 [1.00-1.43]	1.13 [0.93–1.27]	0.007
LDL_C	2.50 [2.01–3.04]	2.51 [2.02–3.04]	2.42 [2.00-3.04]	0.412
ApoA1	1.24 [1.08–1.40]	1.25 [1.09–1.40]	1.21 [1.05–1.37]	0.211
ApoB	0.91 [0.77–1.07]	0.92 [0.77–1.07]	0.86 [0.74–1.04]	0.227
potassium	4.02 [3.76–4.31]	4.02 [3.75–4.31]	4.01 [3.83–4.30]	0.551
tPSA	5.13 [2.07–12.28]	4.49 [1.88–9.70]	80.96 [20.35–100.00]	< 0.001
RBC	4.39 [3.84–4.83]	4.41 [3.89–4.83]	4.19 [3.30–4.75]	0.009
WBC	7.97 [6.36–10.40]	8.02 [6.37–10.56]	7.35 [6.10–9.61]	0.037
HGB	126.00 [111.00-138.00]	127.00 [113.00-138.00]	120.00 [98.50–134.00]	0.004
CRP	8.60 [5.00-35.55]	7.65 [5.00-34.70]	15.64 [5.00-43.37]	0.114
(Median [Q1-Q3])				

Abbreviations: BPH, Benign Prostatic Hyperplasia; PCa, Prostate Cancer; SD, Standard Deviation; Q1-Q3, Interquartile Range; BMI, Body Mass Index; tPSA, Total Prostate-Specific Antigen; LDH, Lactate Dehydrogenase; CK-MB, Creatine Kinase-MB; RBC, Red Blood Cell count

### Feature selection and parsimony

**Fig. 2:**
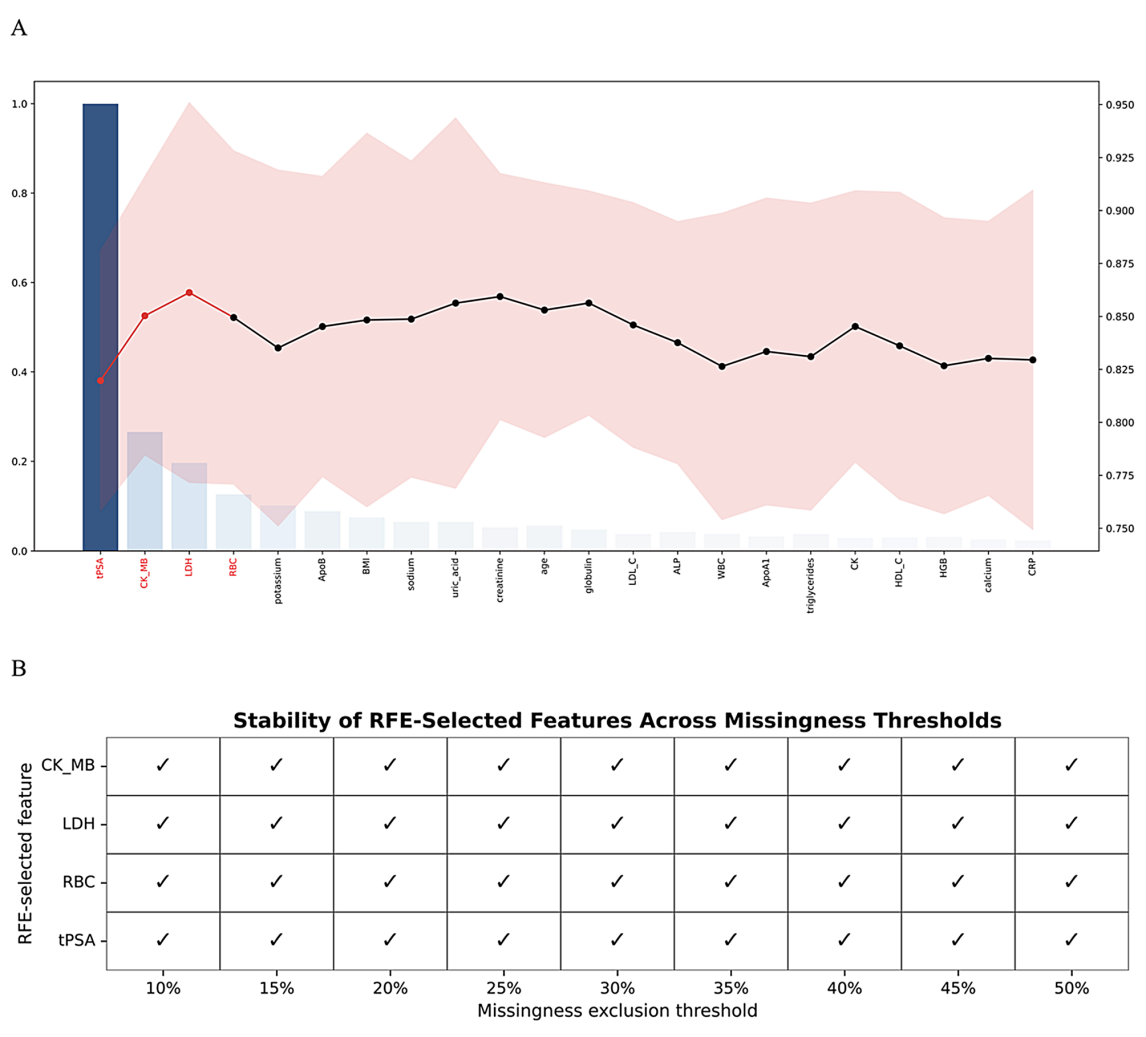
Feature selection and missingness threshold sensitivity analysis. (**A**) Model performance optimization and variable ranking via Recursive Feature Elimination (RFE) based on normalized feature importance scores. (**B**) Sensitivity analysis heatmap illustrating the stability of the RFE-selected feature signature across varying missingness exclusion thresholds (ranging from 10% to 50%)

To maintain model parsimony and avert data leakage, Recursive Feature Elimination (RFE) was rigorously applied exclusively within the training cohort to rank the 22 candidate variables. Subsequent to the ranking procedure, a selection criterion was employed, wherein a normalized feature importance score surpassing a threshold of 0.1 was required**(**Fig. 2A**)**. This thresholding process identified four principal predictors: tPSA, CK-MB, LDH, and RBC. Furthermore, a sensitivity analysis demonstrated that this specific four-biomarker signature remained highly stable across varying missingness exclusion thresholds (ranging from 10% to 50%), confirming that the predefined 30% cut-off did not introduce feature selection bias**(**Fig. 2B**)**. These four features were then utilized in the development of the final machine learning models.

### predictive performance of the models

**Fig. 3:**
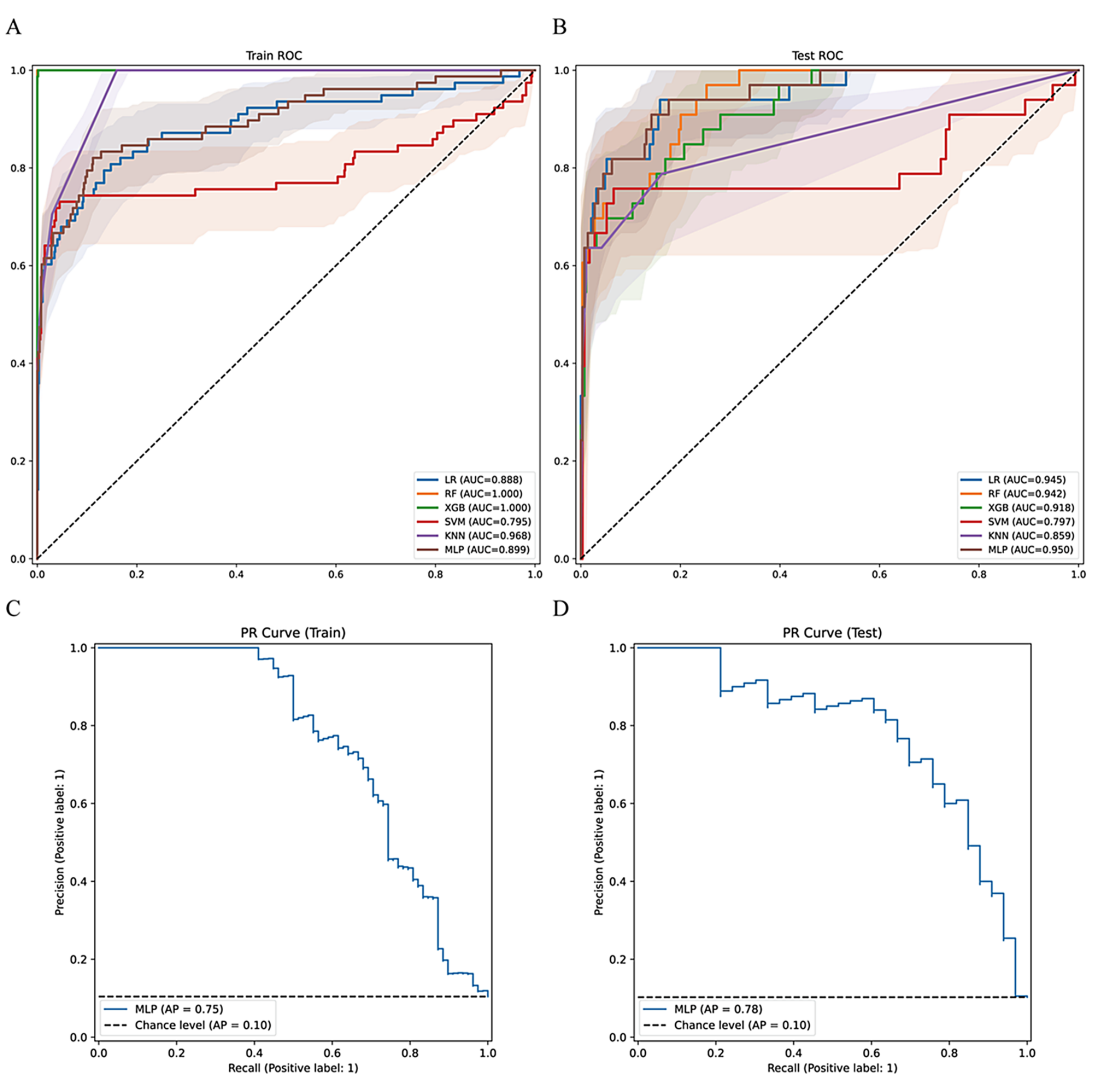
Performance evaluation of machine learning models in the training and test sets. (**A**-**B**) Receiver Operating Characteristic (ROC) curves of the six machine learning algorithms (LR, RF, XGB, SVM, KNN, and MLP) in the training and test cohorts, respectively. (**C**-**D**) Precision-Recall (PR) curves for the optimized Multilayer Perceptron (MLP) model, with the dashed line representing the baseline chance level

The MLP model exhibited strong discriminative performance **(**Table 2**)**, achieving a test AUROC of 0.949 (95% confidence interval [CI]: 0.905 to 0.982). This performance was closely followed by the Logistic Regression (LR) model with an AUROC of 0.946, and the Random Forest (RF) model with an AUROC of 0.943, as depicted in Fig. 3A and B. Furthermore, the Precision-Recall (PR) curve analysis, presented in Fig. 3C and D, provided complementary evidence of model performance in the context of class imbalance, with 10.4% of instances belonging to the positive class.

**Table 2:** Performance metrics of the six machine learning models in the training and test sets

Model	AUC_Train	AUC_Test	ACC_Train	ACC_Test	SENS_Train	SENS_Test	SPEC_Train	SPEC_Test	F1_Train	F1_Test
LR	0.888 (0.832–0.939)	0.946 (0.902–0.983)	0.941 (0.925–0.957)	0.953 (0.932–0.975)	0.503 (0.382–0.615)	0.603 (0.428–0.759)	0.992 (0.985–0.999)	0.993 (0.983-1.000)	0.638 (0.532–0.730)	0.728 (0.585–0.849)
RF	1.000 (1.000–1.000)	0.943 (0.908–0.976)	0.995 (0.989–0.999)	0.953 (0.929–0.975)	0.948 (0.894–0.988)	0.601 (0.421–0.767)	1.000 (1.000–1.000)	0.993 (0.983-1.000)	0.973 (0.943–0.994)	0.726 (0.564–0.852)
XGB	1.000 (1.000–1.000)	0.918 (0.869–0.959)	0.995 (0.989–0.999)	0.950 (0.925–0.972)	0.949 (0.892–0.989)	0.604 (0.440–0.757)	1.000 (1.000–1.000)	0.990 (0.978-1.000)	0.973 (0.944–0.994)	0.712 (0.558–0.840)
SVM	0.795 (0.717–0.870)	0.797 (0.676–0.907)	0.943 (0.927–0.959)	0.953 (0.932–0.975)	0.512 (0.400-0.628)	0.608 (0.432–0.763)	0.993 (0.986–0.999)	0.993 (0.982-1.000)	0.648 (0.549–0.741)	0.723 (0.588–0.844)
KNN	0.968 (0.957–0.979)	0.859 (0.778–0.936)	0.944 (0.927–0.960)	0.953 (0.929–0.972)	0.564 (0.455–0.680)	0.630 (0.444–0.793)	0.988 (0.980–0.996)	0.990 (0.976-1.000)	0.678 (0.579–0.767)	0.733 (0.593–0.853)
MLP	0.899 (0.850–0.939)	0.949 (0.905–0.982)	0.948 (0.932–0.964)	0.953 (0.929–0.975)	0.574 (0.461–0.689)	0.639 (0.471–0.792)	0.991 (0.984–0.997)	0.990 (0.979-1.000)	0.697 (0.601–0.781)	0.734 (0.607–0.857)

Abbreviations: AUC, Area Under the Receiver Operating Characteristic Curve; ACC, Accuracy; SENS, Sensitivity (Recall); SPEC, Specificity; F1, F1-score; LR, Logistic Regression; RF, Random Forest; XGB, eXtreme Gradient Boosting (XGBoost); SVM, Support Vector Machine; KNN, K-Nearest Neighbors; MLP, Multilayer Perceptron

**Fig. 4:**
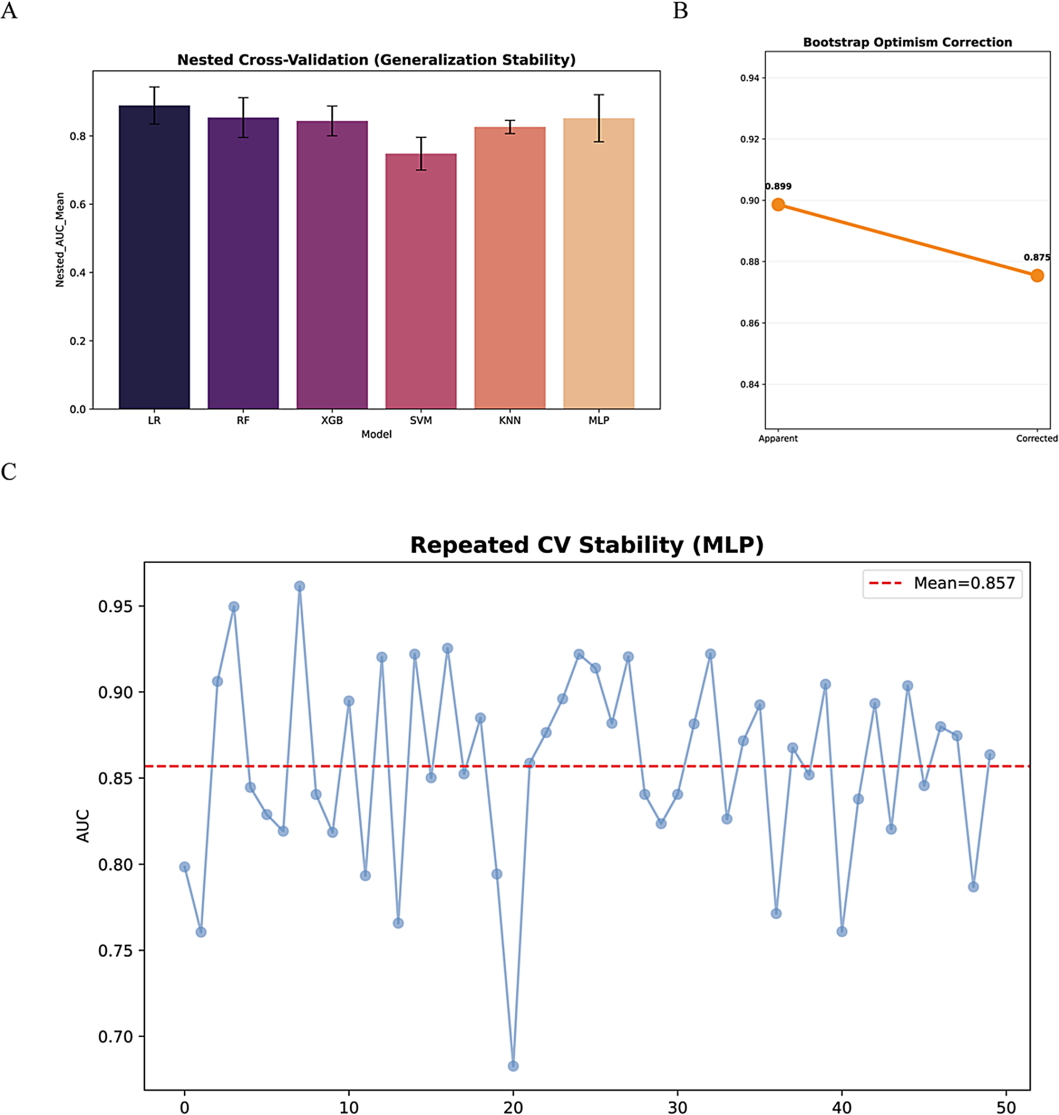
Internal validation and overfitting assessment. (**A**) Nested cross-validation results comparing the discriminative performance across all six evaluated machine learning models. Bars represent the mean Area Under the Receiver Operating Characteristic Curve (AUC), and error bars indicate the standard deviation across validation folds. (**B**) Bootstrap optimism correction analysis for the optimal Multilayer Perceptron (MLP) model, illustrating the relationship between the apparent model performance and the optimism-corrected performance. (**C**) Repeated cross-validation trajectory for the MLP model, displaying the distribution of AUC scores across 50 independent validation iterations (10 repeats of 5-fold cross-validation) to assess algorithmic stability

To address potential optimism in performance estimates and ensure algorithmic stability, internal validation was performed**(**Fig. 4**)**. Initially, nested cross-validation**(**Fig. 4A**)** was utilized to estimate the generalization error across the entire cohort, revealing that both the Logistic Regression (LR) and Multi-Layer Perceptron (MLP) models had mean Area Under the Curve (AUC) values of 0.889 ± 0.054 and 0.852 ± 0.069, respectively. For the optimal MLP model, bootstrap optimism correction (Fig. 4B) was applied to quantify and adjust for potential overfitting; the apparent AUC of 0.899 was adjusted by a slight optimism factor to a more conservative value of 0.875, thereby enhancing the reliability of our risk predictions. Additionally, repeated cross-validation (10 repeats of 5-fold cross-validation) (Fig. 4C) demonstrated the stability of the MLP model, which maintained a mean AUC of 0.857 across 50 validation iterations. Collectively, these results indicate that the observed predictive performance was not solely an artifact of a particular data partition and support the consistency of the model’s internal performance estimates.

### Calibration and clinical utility

**Fig. 5:**
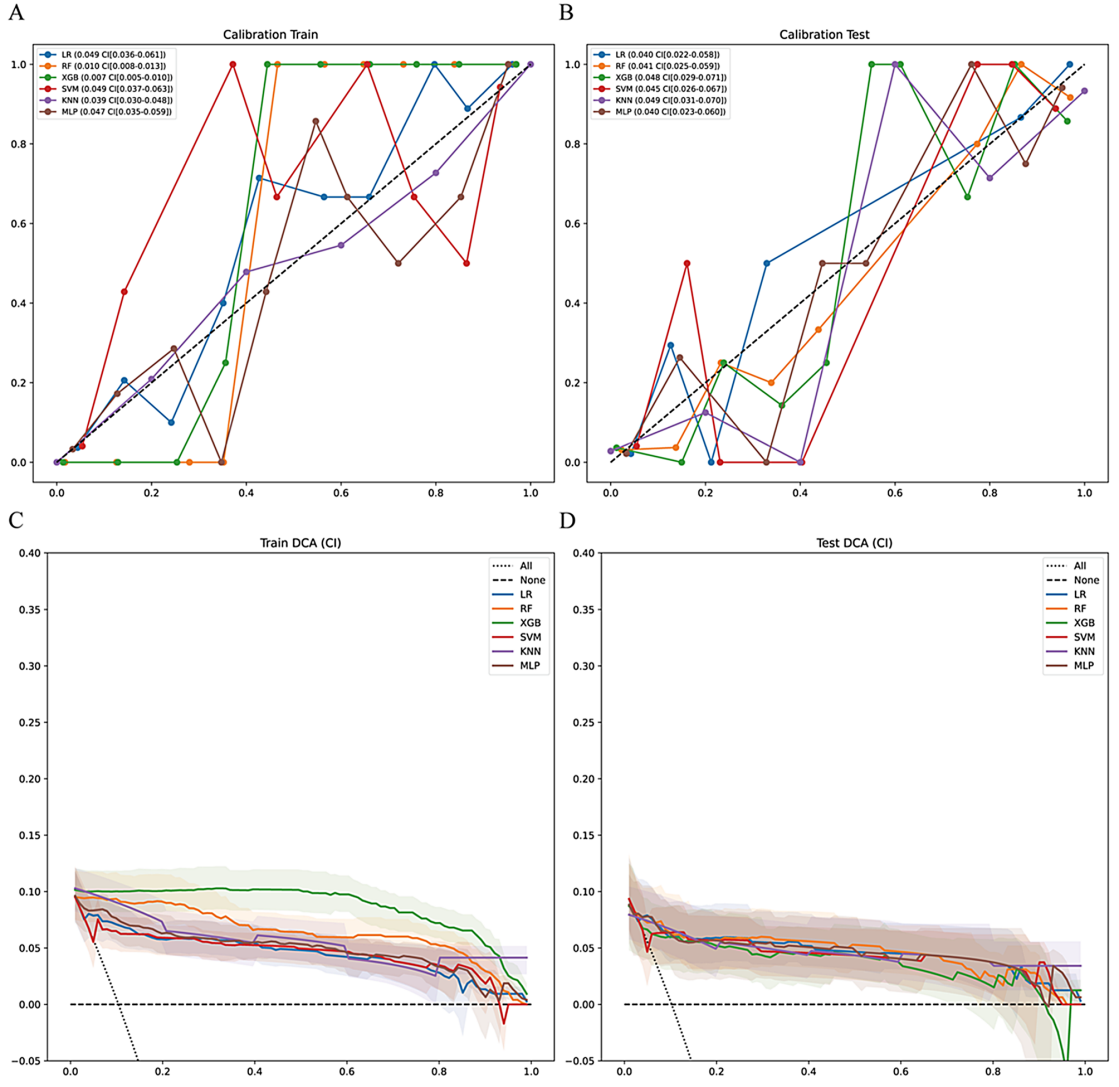
Calibration and clinical utility of the optimized models. (**A**-**B**) Calibration curves of the optimized models in the training set and test set, respectively, comparing predicted probabilities with observed outcomes. The dashed 45-degree line indicates perfect calibration. (**C**-**D**) Decision curve analysis (DCA) of the optimized models in the training set and test set, respectively, demonstrating the clinical net benefit across a range of threshold probabilities compared with the “treat-all” and “treat-none” strategies

The calibration plots**(**Fig. 5A-B**)** suggested good agreement between predicted risks and observed outcomes, as evidenced by low Brier scores ranging from 0.04 for the MLP, LR, RF, and SVM models to 0.05 for the XGB and KNN models. Decision curve analysis **(**Fig. 5C-D**)** indicated that the MLP and LR models provided a positive net benefit across a broad range of threshold probabilities, with the most clinically relevant benefit observed between 0.10 and 0.40.

### Model interpretability via shap analysis

**Fig. 6:**
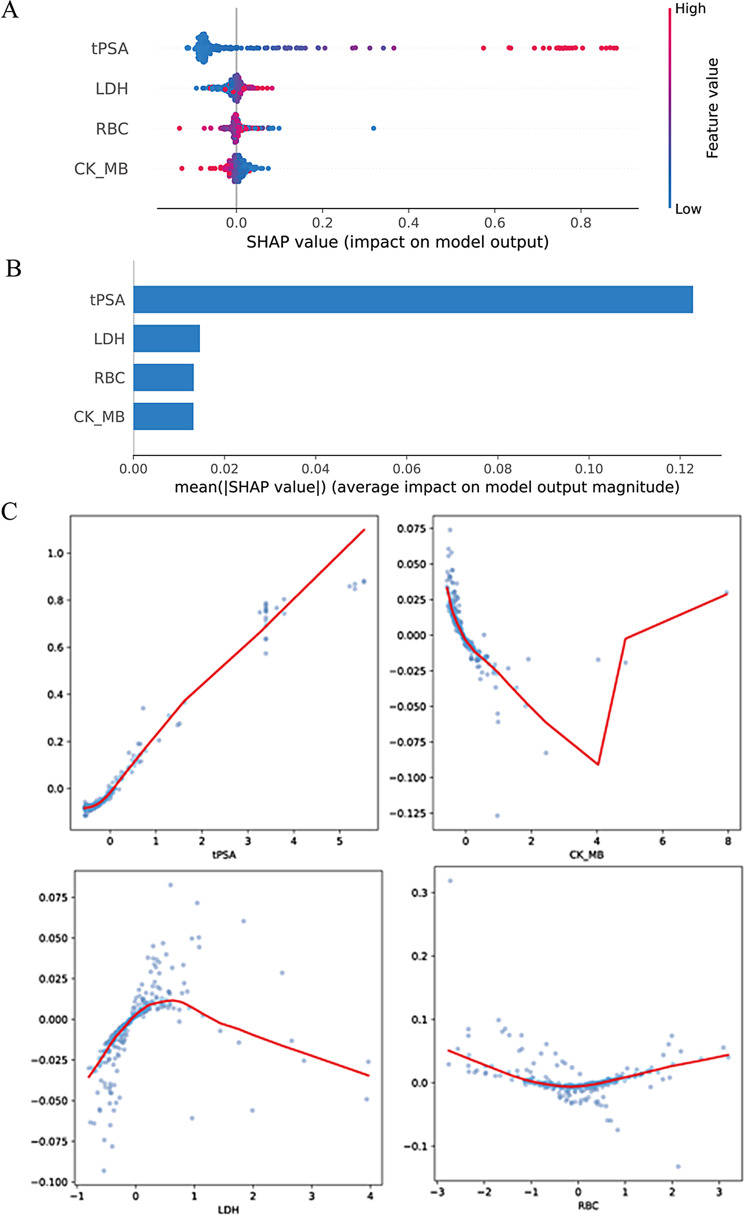
Global and local interpretability of the prediction model via SHAP analysis. (**A**) SHAP summary dot plot illustrating the distribution of the impact of each feature on the model output. Each point represents an individual patient, with colors indicating feature values (red: high; blue: low). (**B**) Global feature importance ranking based on the mean absolute SHAP values. (**C**) SHAP dependence plots with LOWESS

The SHAP summary plots (Fig. 6A) demonstrated the directional influence of each predictor, while the global feature importance ranking (Fig. 6B) quantitatively identified tPSA (mean |SHAP| = 0.123) and LDH (mean |SHAP| = 0.015) as the most significant determinants of risk. These were closely followed by RBC (mean |SHAP| = 0.013) and CK-MB (mean |SHAP| = 0.013). Additionally, the LOWESS analysis of SHAP values (Fig. 6C) revealed a pronounced, nonlinear increase in the probability of prostate cancer (PCa) as tPSA levels surpassed critical thresholds. Lower RBC counts and higher CK-MB levels were also clearly associated with increased SHAP values.

### Model performance in the psa gray zone

**Fig. 7:**
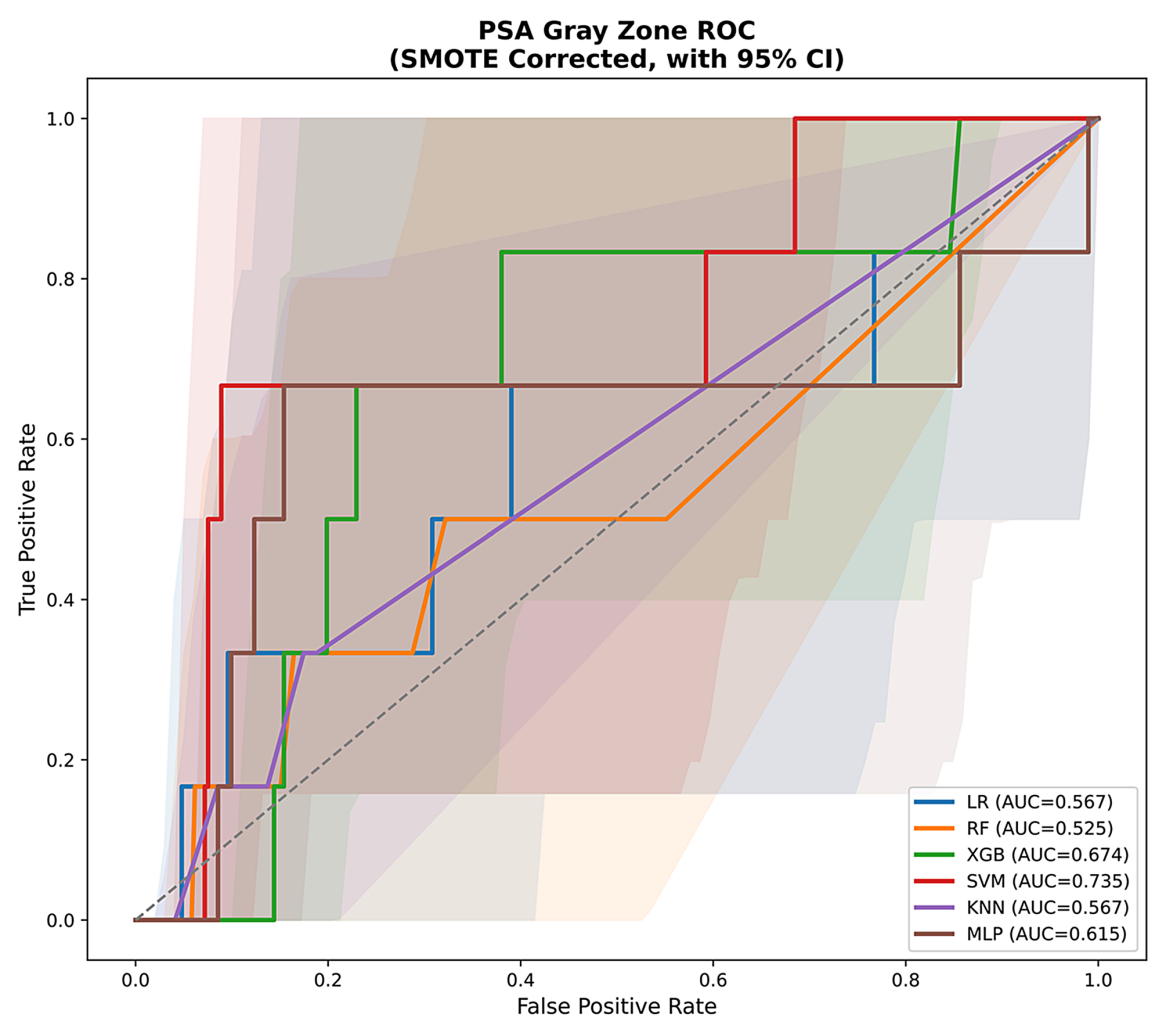
ROC curves for the six evaluated models within the PSA gray zone (4.0 to 10.0 ng/mL)

An analysis was conducted on the sub-cohort of patients within the PSA gray zone (4.0 to 10.0 ng/mL, *N* = 296) to explore model behavior in this diagnostically ambiguous subgroup. To mitigate the extreme class imbalance (6 latent PCa cases out of 296 patients) and prevent overoptimistic fitting, SMOTE was integrated into the validation pipeline. Under these resampling-adjusted conditions, the support vector machine (SVM) model achieved an AUC of 0.739; however, the extremely wide 95% confidence interval (0.490 to 0.936) indicates profound uncertainty in its predictive capacity. Furthermore, the sensitivity (0.674, 95% CI: 0.250 to 1.000) and other evaluation metrics, including the area under the precision-recall curve (AUPRC), remained poor across all classifiers **(**Table 3; Fig. 7**)**. These findings underscore that the risk stratification in this gray-zone subgroup is fundamentally data-limited, rather than indicative of specific algorithmic robustness. Consequently, predictive performance in this highly imbalanced clinical scenario remains highly uncertain.

**Table 3:** Performance metrics of the six machine learning models in the PSA gray zone (4.0 to 10.0 ng/mL) sub-cohort following SMOTE correction

Model	AUC(95% CI)	Sensitivity(95% CI)	Specificity(95% CI)
LR	0.571 (0.264–0.867)	0.337 (0.000-0.800)	0.699 (0.647–0.749)
RF	0.531 (0.225–0.842)	0.000 (0.000–0.000)	0.962 (0.939–0.983)
XGB	0.680 (0.447–0.848)	0.000 (0.000–0.000)	0.938 (0.910–0.963)
SVM	0.739 (0.490–0.936)	0.674 (0.250-1.000)	0.911 (0.878–0.942)
KNN	0.570 (0.395–0.815)	0.171 (0.000-0.600)	0.863 (0.823-0.900)
MLP	0.621 (0.262–0.907)	0.000 (0.000–0.000)	0.942 (0.914–0.966)

Note: Metrics are reported with 95% Confidence Intervals (CI) derived through bootstrap resampling. To address the severe class imbalance within this subgroup (only six incident PCa cases), the Synthetic Minority Over-sampling Technique (SMOTE) was incorporated into the cross-validation pipeline. This resampling strategy was intended to reduce mathematical artifacts, such as perfect separation, that may occur in uncorrected models under severe class imbalance. Nevertheless, given the scarcity of positive cases, these metrics should be interpreted conservatively as exploratory estimates of algorithmic behavior in this highly ambiguous clinical subgroup

### Deployment of the web-based prediction application

**Fig. 8:**
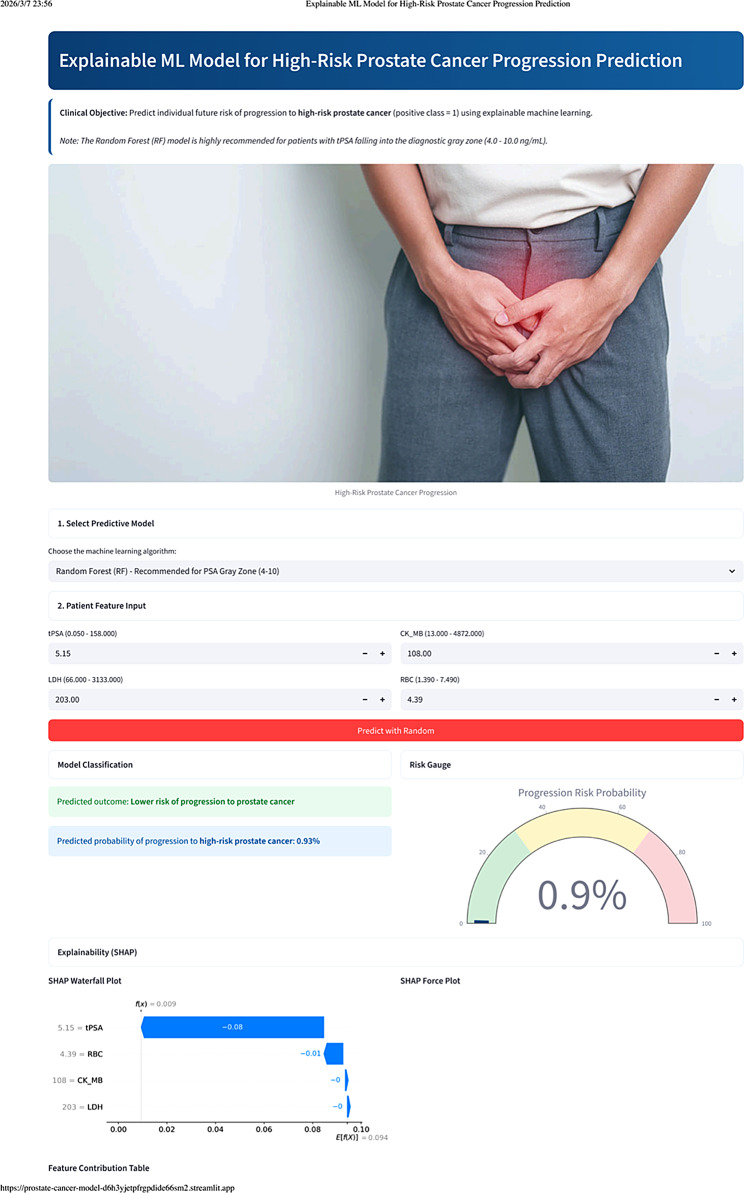
User interface of the web-based prediction application

To facilitate clinical visualization, a web-based application was developed using Streamlit (https://prostate-cancer-model-d6h3yjetpfrgpdide66sm2.streamlit.app/). This interactive platform serves as an accessible online tool for assessing the risk of prostate cancer (PCa) in patients initially diagnosed with benign prostatic hyperplasia (BPH). To translate the subgroup findings into the web interface, the platform incorporates the top-performing general model (MLP) but does not present any gray-zone subgroup model as clinically reliable. If a patient’s tPSA falls within the diagnostic gray zone (4.0 to 10.0 ng/mL), the application automatically issues a cautionary notification indicating that predictions in this range are exploratory, data-limited, and should be interpreted with extreme caution. By inputting the four selected clinical features—total prostate-specific antigen (tPSA), lactate dehydrogenase (LDH), creatine kinase-MB (CK-MB), and red blood cell count (RBC)—into the designated fields, clinicians can instantly receive individualized prediction probabilities along with a personalized SHAP waterfall plot for enhanced clinical transparency. The platform was developed using the Streamlit framework with a backend implemented in Python. It is crucial to emphasize that this tool is designed solely for research purposes and does not constitute a certified medical device **(**Fig. 8**)**.

## Discussion

Over the past several decades, the widespread adoption of PSA screening has altered the epidemiological profile of prostate cancer. Recent analyses utilizing frailty models on large population registries indicate that the application of PSA testing effectively reduces the pool of susceptible individuals at progressively younger ages [18, 19]. As a result, clinical practice is increasingly confronted with older men diagnosed with BPH who experience considerable diagnostic uncertainty. This subpopulation often harbors occult cancer that conventional PSA thresholds fail to stratify accurately [5, 20]. Because our framework is designed to identify these pre-existing, occult cancers missed during initial assessments, rather than to forecast de novo carcinogenesis, it functions primarily as a triage tool for early detection. The biomarker signature reflects pathophysiological changes associated with latent tumors, precluding its application for predicting future oncogenesis in individuals with genuinely benign prostates. The epidemiological challenge is further compounded by the biological reality that prostate cancer (PCa) is a multifocal disease characterized by significant intratumoral heterogeneity and a highly intricate genomic landscape [2]. To address this critical diagnostic issue, our study sought to bridge the translational gap between routine clinical data, epidemiological susceptibility patterns, and advanced machine learning techniques. Our optimal machine learning framework achieved a test AUROC of approximately 0.95, suggesting promising internal discriminative ability to differentiate between stable BPH and latent PCa, a clinical scenario in which conventional PSA-based risk calculators often lack specificity [21, 22]. Although established clinical tools, such as the European Randomized Study of Screening for Prostate Cancer (ERSPC) risk calculator and the Prostate Imaging Reporting and Data System (PI-RADS), are widely endorsed [23, 24], our model is designed to complement them by occupying a pre-imaging triage niche. The retrospective nature of our initial cohort of patients with benign prostatic hyperplasia (BPH) limited our ability to perform a direct quantitative comparison using comprehensive multiparametric MRI (mpMRI) PI-RADS scores and precise transrectal ultrasound measurements of transition zone volumes. Nonetheless, existing literature suggests that risk calculators predominantly developed from Western cohorts, such as the European Randomized Study of Screening for Prostate Cancer (ERSPC) or the Prostate Cancer Prevention Trial (PCPT), may overestimate the risk of prostate cancer (PCa) in Asian populations. This overestimation is attributed to inherent ethnic differences in baseline prostate volume and prostate-specific antigen (PSA) density [25]. Moreover, while previous research has extensively utilized costly genomic risk scores or resource-intensive MRI-based radiomics [26], the broad adoption of multiparametric MRI as a primary screening tool is often hindered by its high cost and the need for specialized radiological expertise [24]. In this context, by utilizing readily accessible and routinely collected blood markers, our model may serve as a useful adjunct for risk stratification as a cost-effective preliminary triage step prior to imaging. It is designed to identify high-risk patients who would derive the greatest benefit from subsequent multiparametric MRI (mpMRI) or biopsy assessments, thereby optimizing personalized clinical management and minimizing the incidence of unnecessary invasive procedures.

The model’s performance was further evaluated within the diagnostically ambiguous PSA gray zone (4.0 to 10.0 ng/mL, *N* = 296). To rigorously address the issue of extreme class imbalance and prevent overfitting to the majority class due to data scarcity, we employed SMOTE. Under these adjusted conditions, the predictive performance in the PSA gray zone remained poor and highly uncertain, as evidenced by the extremely wide confidence intervals (e.g., SVM AUC: 0.739, 95% CI: 0.490 to 0.936) and suboptimal precision-recall metrics. Rather than demonstrating the robustness of a specific algorithm, this evaluation highlights a fundamentally data-limited problem driven by the extreme scarcity of positive cases (*N* = 6) in this subgroup. Therefore, algorithmic predictions for patients presenting within this range lack reliability and should not be used to guide clinical decisions without further validation in larger, balanced cohorts.

In addition to assessing discriminative accuracy, it is crucial to evaluate the practical clinical utility of a predictive model. Decision Curve Analysis (DCA) revealed that our framework consistently provided a higher net benefit compared to the default strategies of ‘treat all’ and ‘treat none.’ This net benefit was particularly significant within the threshold probability range of 10% to 40%. As initially proposed by Steyerberg and Vickers [27] and subsequently applied in the validation of established risk calculators [23], this threshold range is widely recognized as the optimal decision zone for recommending prostate biopsies in contemporary clinical practice. A positive net benefit within this range supports the clinical utility of the model. Clinically, implementing our framework to guide decision-making within the 10–40% range could enable clinicians to avoid a substantial number of unnecessary invasive procedures or costly imaging, while effectively minimizing the risk of overlooking occult malignancies.

The identification of LDH, RBC, and CK-MB as principal predictors alongside tPSA is of significant biological interest, as it highlights a complex, multidimensional signature of the tumor microenvironment that conventional PSA testing fails to capture. The initiation and progression of prostate cancer (PCa) are driven by distinct molecular events, such as PTEN loss, hyperactivation of the PI3K/AKT pathway, and MYC amplification [3]. These genomic alterations compel cancer cells to undergo metabolic reprogramming, commonly referred to as the Warburg effect. Within this framework, LDH is not merely a general marker of tissue damage; rather, it is a pivotal enzyme in the anaerobic glycolytic pathway and acts as a direct, systemic indicator of high cellular turnover, intraprostatic hypoxia, and tumor-induced metabolic stress [15, 28]. Nonetheless, it is important to recognize that LDH functions as a systemic associative marker rather than a direct causal factor in prostate oncogenesis. In this cohort of elderly patients with benign prostatic hyperplasia (BPH), elevated levels of lactate dehydrogenase (LDH) may indicate a broader burden of comorbidities, subclinical tissue injury, or other metabolic processes not specific to cancer. The selection of LDH by the machine learning model is hypothesized to computationally capture these underlying metabolic pathways.

Furthermore, the selection of RBC count and CK-MB reflects the systemic footprint of early-stage PCa rather than a localized effect. The inverse correlation between RBC counts and PCa risk is consistent with our baseline findings **(**Table 1**)**, which show that patients diagnosed with PCa also had lower median hemoglobin levels (120.0 vs. 127.0 g/L). This trend supports the hypothesis that cancer-induced systemic inflammation and alterations in the hematological milieu, potentially leading to anemia of chronic disease, often precede clinical metastasis [29, 30]. Early malignancies may secrete pro-inflammatory cytokines that cause mild bone marrow suppression or anemia of chronic disease, serving as indicators of a systemic response to the tumor. With respect to CK-MB, its conventional role as a cardiac-specific enzyme, rather than a standard oncological biomarker, necessitates careful interpretation of its selection by our algorithm. Nonetheless, its identification as a robust predictor is biologically plausible when considered in the context of tumor metabolic reprogramming. Recent mechanistic studies underscore the critical involvement of the broader creatine kinase family in cellular bioenergetics, particularly in sustaining ATP homeostasis in rapidly proliferating cancer cells [31, 32]. As premalignant prostatic lesions advance, the localized microenvironment often encounters significant metabolic stress, characterized by hypoxia and nutrient deprivation. To endure and maintain a high proliferative capacity under these adverse conditions, malignant cells substantially upregulate the phosphocreatine creatine kinase shuttle system [32]. Consequently, the CK-MB signal observed in our cohort is posited not as a mere statistical anomaly but as a systemic surrogate marker indicative of the intense cellular energy metabolism and stress within the prostatic microenvironment. This hypothesis is consistent with reports of elevated CK-MB ratios in various malignancies [33], suggesting that standard automated assays may detect atypical, tumor-derived CK fractions or reflect the systemic metabolic burden associated with early carcinogenesis. Until independent biological validation is performed, the CK-MB signal in this computational model should be interpreted cautiously as an associative surrogate representing a broader metabolic burden rather than a direct causal mechanism. The precise biological function of this biomarker in prostate cancer warrants additional fundamental research. Collectively, this four-biomarker signature provides a comprehensive overview of prostate-specific signaling (tPSA), metabolic reprogramming (LDH), and systemic host response (RBC and CK-MB), serving as a cost-effective decision-support tool.

Our findings closely align with the “Unified Prediction Model” concept proposed by Aly et al. [21]. Nevertheless, our findings extend this framework by suggesting that promising internal predictive performance can be attained through the AI-driven integration of routine laboratory markers, even in the absence of multiparametric MRI or costly genomic sequencing panels [34]. A significant obstacle to clinical AI implementation is the opaque nature of deep learning algorithms [13]. Furthermore, the substantial variability in individual cancer susceptibility dictates that risk rarely increases in a simple linear manner [19], necessitating the use of complex, non-linear models whose internal decisions must be interpretable. By employing SHAP, our framework addresses these transparency concerns and enhances clinical trust. The interpretability of our model through SHAP values exemplifies a broader movement towards knowledge-augmented AI in healthcare. Although our model relies on post hoc explanations, its emphasis on a succinct biomarker signature is consistent with structured hybrid modeling strategies that concentrate explainability on clinically relevant substructures or clusters [12]. Furthermore, emerging paradigms suggest that model interpretability may be enhanced by the reconstruction of clinical networks utilizing large language models (LLMs), which align machine learning outputs with clinical expertise [35]. The LOWESS curves generated in our study illustrate nonlinear statistical thresholds, exemplified by the exponential increase in risk observed when tPSA and LDH exceed specific limits within this cohort. Although these visualizations provide valuable insights into the model’s decision-making process, SHAP values and LOWESS trajectories inherently represent post hoc computational associations rather than definitive biological causality. As a result, these visual interpretations primarily serve as data-driven heuristics, highlighting specific clinical phenotypes associated with increased prostate cancer risk. Ultimately, translating these algorithmic patterns into established biological mechanisms will necessitate subsequent validation through independent clinical cohorts and targeted experimental studies.

While the model demonstrated promising internal performance, this study is subject to several critical limitations. Foremost among these is the retrospective, single-center design, which introduces inherent spectrum bias. The model’s performance is strictly dependent on the specific laboratory measurement protocols, assay instrumentation, and patient demographics of a single institution. Consequently, the high AUC values reported in this study are likely optimistic, and the generalizability of the four-biomarker signature is currently restricted. We explicitly emphasize that the immediate clinical applicability of this framework is negligible. The clinical potential of this signature cannot be realized without external, multi-center validation, which remains the most critical barrier to its clinical translation. Future studies must be conducted in independent, external cohorts from diverse healthcare settings using harmonized laboratory protocols and standardized operating procedures (SOPs) to rigorously assess performance. Furthermore, the implementation of stringent exclusion criteria, such as a history of malignancy or active prostatitis, likely introduced selection bias, potentially skewing the cohort towards individuals with a healthier baseline. This limitation constrains the generalizability of the findings to real-world patients with benign prostatic hyperplasia (BPH) who present with complex comorbid conditions. Moreover, the absence of data on unmeasured confounders, including socioeconomic factors and comprehensive medication histories beyond 5-ARI therapy, limits our findings to associative correlations rather than establishing definitive causal relationships. Additionally, both the incidence rates and the inherent biological susceptibility to prostate cancer demonstrate significant ethnic, genetic, and geographic variations [36]. To move beyond clinical associations and assess applicability in real-world settings, future validations should intentionally include a more diverse population of individuals with BPH, encompassing those with varying levels of baseline inflammation and complex comorbidities. To advance beyond clinical correlations, future research should also employ sophisticated preclinical models, such as patient-derived organoids and patient-derived xenografts [9, 10], to mechanistically elucidate the interactions between these metabolomic profiles and the prostate tumor microenvironment. Ultimately, the proposed framework serves primarily as an exploratory, hypothesis-generating tool. Therefore, the associated online platform is designed for research purposes, pending further prospective validation.

## Conclusion

In conclusion, this study developed and internally validated an interpretable machine learning framework utilizing a four-biomarker signature to identify latent PCa in patients initially diagnosed with BPH. By integrating routine laboratory data with advanced analytical techniques, this framework demonstrated the potential to improve risk stratification within this retrospective single-center cohort, but its diagnostic value remains exploratory until validated in independent external cohorts. The introduction of our online risk assessment platform serves as an accessible tool currently designated for research purposes. Subject to prospective validation, this framework establishes a conceptual basis for future decision support strategies aimed at optimizing individualized clinical management and minimizing unnecessary invasive procedures.

## Data Availability

The datasets used and/or analysed during the current study are available from the corresponding author on reasonable request. The web application for the best-performing prediction model is publicly available at: (https://prostate-cancer-model-d6h3yjetpfrgpdide66sm2.streamlit.app/). To ensure computational reproducibility and transparency, the complete Python source code utilized for data preprocessing, model training, internal validation, and interpretation in this study has been open-sourced and is publicly available on GitHub: (https://github.com/Joeaicool/python-code-backup).
